# Introducing Social Breathing: A Model of Engaging in Relational Systems

**DOI:** 10.3389/fpsyg.2021.571298

**Published:** 2021-04-08

**Authors:** Niclas Kaiser, Emily Butler

**Affiliations:** ^1^Department of Psychology, Faculty of Social Sciences, Umeå University, Umeå, Sweden; ^2^Family Studies and Human Development, University of Arizona, Tucson, AZ, United States

**Keywords:** non-verbal behavior, implicit processes, shared intentionality, non-linear dynamics, mutual regulation, multi-brain networks, relational systems

## Abstract

We address what it means to “engage in a relationship” and suggest Social Breathing as a model of immersing ourselves in the metaphorical social air around us, which is necessary for shared intention and joint action. We emphasize how emergent properties of social systems arise, such as the shared culture of groups, which cannot be reduced to the individuals involved. We argue that the processes involved in Social Breathing are: (1) automatic, (2) implicit, (3) temporal, (4) in the form of mutual bi-directional interwoven exchanges between social partners and (5) embodied in the coordination of the brains and behaviors of social partners. We summarize cross-disciplinary evidence suggesting that these processes involve a multi-person whole-brain-body network which is critical for the development of both we-ness and relational skills. We propose that Social Breathing depends on each individual’s ability to sustain multimodal interwovenness, thus providing a theoretical link between social neuroscience and relational/multi-person psychology. We discuss how the model could guide research on autism, relationships, and psychotherapy.

Extensive evidence suggests that there is something important going on between people that cannot be reduced to the individuals themselves (Anderson, 2009; Reddy and Uithol, 2016; Szanto and Krueger, 2019). Numerous models have been developed in an attempt to capture this elusive “something” but none are completely satisfying. We therefore build on previous attempts and present Social Breathing, which is a cross-theoretical synthesis highlighting consensus between models from a variety of scientific domains. As shown in Figure 1, we suggest the latent construct of Social Breathing (e.g., a theoretical entity that cannot be measured directly) to indicate a fundamental human activity that cannot be accomplished by an individual alone. When we are Socially Breathing, we are immersed in a multi-person system, meaning we are fully engaged in a social interaction, including sharing intentions, joint meaning-making and complex coordination. Given the centrality of Social Breathing for human functioning, it is not surprising that many scientific domains have tried to understand it. But as suggested in Figure 1, each domain we are aware of captures some aspects of Social Breathing, but not all. Our model is not new in any of its details, therefore, but it brings together existing concepts from disparate fields and integrates them under the concept of Social Breathing, thereby fleshing out a description of the process and making it easier to develop specific and testable hypotheses.

**FIGURE 1:**
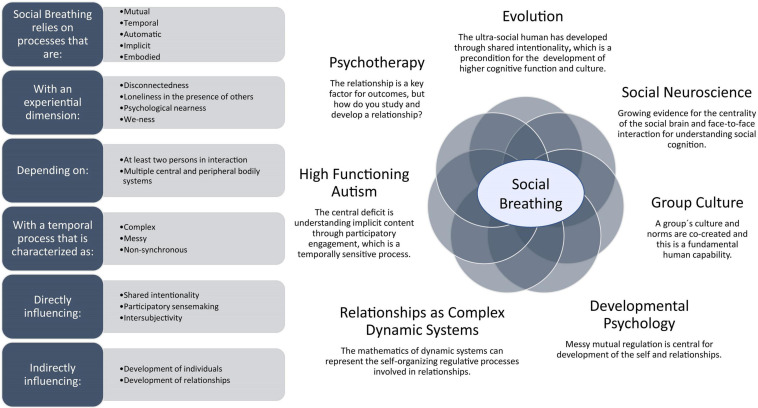
Social Breathing is a latent construct (e.g., a theoretical entity that cannot be measured directly) representing the fundamental human activity of becoming engaged in a social interaction, including sharing intentions, joint meaning-making, and complex coordination. We list its characteristics in the panel on the left. Numerous research areas attempt to capture this elusive “something” but none address all aspects of Social Breathing. On the right we show a representative, but not exhaustive, set of such research domains. We use Social Breathing to synthesize across domains, making it easier to develop specific and testable hypotheses.

In particular, there has been a shift in the social neuroscience of relationships, with a renewed emphasis on persons co-creating relational systems during interaction with each other (Cooke et al., 2013; Szanto and Krueger, 2019). Research areas as diverse as social (Butler, 2011; Ferrer and Helm, 2013) and developmental psychology (Stern, 2000; Reddy and Uithol, 2016), group dynamics (Wheelan, 2005) and team performance (Cooke et al., 2013), neuroscience (Di Paolo and De Jaegher, 2012; Hari, 2017), and affective science (Damasio, 1999; Balconi and Fronda, 2020) recognize that relationships are more than the sum of the individuals involved and need to be understood at this non-individualistic level of analysis. Our model joins this expansion on individualistic approaches by emphasizing the interwoven character of relational systems, where interwovenness is defined as a highly complex coupling of multiple bodies (including brains) and behaviors, characterized by diverse temporal dynamics across multiple modalities.

We review work from a number of different scientific domains, each of which addresses some aspects of Social Breathing, with contributions from evolutionary theory (Tomasello et al., 2012; Tomasello, 2014), social neuroscience (Di Paolo and De Jaegher, 2012; Hari et al., 2015), clinical work on autism (Du Bois et al., 2014; Gallese and Rochat, 2018), mathematical modeling of social dynamics (Butler, 2011; Tognoli et al., 2020), developmental psychology (Stern, 1990, 2004, 2010; Tronick and Beeghly, 2011), psychology and culture of groups (Wheelan, 2005; Cooke et al., 2013) and psychotherapy (Stern et al., 1998; Gelso, 2009; Norcross and Lambert, 2014). Based on our review, we highlight the relational process of interwovenness and argue for its centrality in the emergence of shared intentionality, which refers to when two or more people experience and understand the world almost as if they were one person (Tomasello, 2014). We introduce the model of Social Breathing to explain how interwovenness might work, emphasizing the fact that we are engaged in life-supporting participatory meaning-making, which is necessary for shared intentionality. We argue that the process that enables us to establish shared intentionality is not like a tennis match, as implied by sequential models of interaction, where a sender codes a message and a receiver decodes it (Hari, 2017). Rather, it involves being intertwined in complex ways with others at multiple levels (Szanto and Krueger, 2019).

A variety of theories exist about the processes underlying shared intentionality, all of which include at least the following five tenets: They are (1) non-linguistic, (2) automatic (e.g., do not require conscious control), (3) reciprocal between social partners (e.g., they cannot be reduced to an individual) (4) temporally dynamic, and (5) must be embodied in the coordination of the brains and behaviors of social partners. In line with the writing of Hari (2017), we propose that it should be possible to study this dynamic multi-person system with novel experimental designs, hyperscanning (i.e., simultaneous visualization of multiple brains) and mathematical modeling, to address the question of “how do the bodies (including brains) and behaviors of a dyad or social group work?” Such research will have implications for understanding a diverse set of domains, including autism, group dynamics and team performance, psychotherapy, family systems, professional development for persons in caring and leadership professions, social artificial intelligence (AI), and human-computer interaction.

## Engaging in Relational Systems

It is uncontroversial to argue that humans are fundamentally social and that the human brain has evolved for survival in social contexts. Any interdependent group of people form a relational system, with emergent properties that cannot be reduced to the individuals involved. In particular, patterns of interaction emerge automatically, with self-sustaining organization and a momentum of their own (De Jaegher et al., 2010). In other words, whenever people come together they form a system functioning as a whole (Krueger, 2013). Such relational systems have rules, or norms, regulating what may be done within the group, what different expressions mean, and what sanctions mark that someone is out of bounds. In order to function together we need each individual to develop basic competencies in navigating and negotiating the groups’ agreements. In other words, a relational system requires its members to develop at least a basic level of social skills, especially the ability to respond appropriately to shared meanings and to sustain interaction (De Jaegher et al., 2010).

Relational systems, collective meaning-making and their neural underpinnings are being studied across multiple scientific domains, including evolutionary psychology, social neuroscience, computational social psychology, relational psychology, humanistic psychology and research on psychotherapy and autism. One shared focus across these perspectives is on social processes that are at least partly automatic. For example, research on “shared realities,” which refers to our ubiquitous tendency to perceive commonalities with others’ inner states, emphasizes the automaticity with which we experience a shared reality with others (Echterhoff et al., 2009). Similarly, phenomenological explanations of how we understand others’ inner states emphasize that we automatically assume other people have minds and immediately interpret their behaviors as expressive or intentional (Zahavi, 2011). Or as another example, the Interactive Brain Hypothesis emphasizes that during social interactions the brain may be less involved in purposefully reconstructing the “mental state” of others, and more involved in automatically participating in a dynamic social process outside its full control (De Jaegher et al., 2010).

Another shared focus is on implicitness and non-linguistic communication, meaning that the social processes involved happen beyond the words’ explicit meanings (Frith and Frith, 2008; Tomasello, 2014; Di Cesare et al., 2017; Balconi and Fronda, 2020). Thus, a central premise is that successful engagement in relational systems requires automatic and implicit sharing of social content, including (but not limited to) emotions, assumptions and cognitive representations such as beliefs or values. Shared intentionality provides a prototypical example of the implicitness of relational systems, where two or more persons understand the world more or less as one, as a plural subject (Tomasello, 2014). Shared intentionality enables us to participate with others in collaborative activities and often occurs largely through non-linguistic means, such as pointing, gaze-following, or automatically coordinating behaviors needed to accomplish a task (Tomasello, 2014). Shared intentionality comes with the possibility of experiencing that something is being shared, or at least the competence to micro-adjust actions to make sure that the focus remains shared. The shared object may be the focus of gaze, but also other things, such as the emotion of anger, or a relational awareness such as knowing that things are working fantastically between us, or conversely that our relationship is breaking up and there is nothing we can do about it.

Another premise is that the implicit sharing process is temporal and bidirectional between multiple people (De Jaegher et al., 2010). One person is not sharing with a passive object, but instead a mutual dynamic process is occurring, whereby partners make micro-adjustments over time, driven by implicit information from high-resolution perceptions of the others’ states and intentions (Schore, 2015). Mutual interaction is thus not characterized by an exact mirroring of the other, but rather involves a complex fitting-together of the individuals involved (Reddy and Uithol, 2016), producing a resonance between two attuned systems and feelings of psychological closeness (Schore, 2015), being taken up in a flow, and being connected with the other (De Jaegher et al., 2010). This process has sometimes been referred to as co-regulation, in order to distinguish complex relational systems from linear models where one person influences the other in a unidirectional way (Sbarra and Hazan, 2008; Butler and Randall, 2013). In summary, across a wide range of research domains, there is growing consensus that social relationships depend on engagement in relational systems involving automatic, implicit and mutual sharing of diverse social content and that this ability is likely hard-wired at the neural level due to its evolutionary centrality.

## The Model of Social Breathing

We use the term Social Breathing to refer to when a multi-person system becomes interwoven through the automatic, implicit, temporal and mutual sharing of social content. The model highlights both the multi-person process itself and the individual abilities that are necessary for engaging in it, as well as the experiential aspect of being interwoven with others. For example, when breathing works and the air is good enough, breathing goes on unnoticed. However, we clearly recognize when we are out of air, or that the air lacks oxygen, or that it is contaminated in some way. The same with Social Breathing. We are naturally highly sensitive to the disconnectedness that occurs when a partner is occupied with a smartphone, for example. Conversely, we can identify if the air is better than average. We may revel in the shared joy of close collaboration or an intimate conversation. These experiential aspects of Social Breathing are akin to the idea of Affective Atmospheres (Anderson, 2009), whereby individual experiences both give rise to, and are enveloped by, an affective atmosphere that is immediately tangible, but exists somewhere in-between and around the individuals involved. For example, as soon as we enter a room we can sense a tense or friendly social atmosphere. Anderson also appeals to the metaphor of breathing, saying “… it (the atmosphere) exerts a force on those that are surrounded by it, and like the air we breathe it provides the very condition of possibility for life” (Anderson, 2009, pg. 78).

Another aspect of Social Breathing is that we have the ability to breathe “by will” but as soon as attention is shifted, the life supporting activity goes back to its automatic functionality. As with physical breathing, Social Breathing is fundamental for the evolution of human life, given our complex social nature. The absence of adequate air has far-reaching negative consequences for the individual, just as the necessity for high-quality social “air” partly answers the question of “why people so strongly seek emotional contact and intersubjectivity, and why failure to reach contact has such a damaging effect on infants” (Tronick, 2007). Taking the analogy further, just as we cannot explain extracting oxygen from air by investigating only the contraction of lungs, we hypothesize that Social Breathing will not be understood by focusing on isolated functions, such as language considered without the context of other social behaviors. Although such behaviors are likely key aspects of the process, just as contracting lungs are important for physical breathing, they are not sufficient for explaining its life-supporting nature.

We hypothesize that Social Breathing requires at least two people who have the individual abilities for engaging in it. Further, we expect that these abilities range from inability to high-level ability, with normally distributed between-person variation. As with physical breathing, we further hypothesize that Social Breathing requires typical biological functionality, and when this function is disturbed because of biological or social reasons, there will be negative consequences. We also expect that individual differences in the ability for Social Breathing, like breathing air, are dependent on genetic pre-requisites, practice, and situational circumstances. For example, stress or anxiety should negatively affect the ability, while ambition and interest would increase it. Analogous to physical breathing, some people wish to socially breathe better for private reasons (e.g., improving a failing romantic relationship), and for professional reasons (e.g., being a successful psychotherapist). Finally, as with training athletes to maximize their breathing efficiency, it should be possible to develop and assess empirically supported training programs to enhance Social Breathing in order to intervene in socio-emotional disorders such as autism, to promote wellbeing, and to optimize performance in relevant professions such as psychotherapy.

## Both Individuals and Relationships Develop Through Social Breathing

Since interwoven interaction and shared intentionality are central aspects of human life, they inevitably play a role in the development of both individuals and relationships. In keeping with this idea, various theoretical frameworks in developmental psychology such as open-system theory (Tronick, 2007) and self-psychology (Stern, 2000) have focused on the biopsychosocial, mutual and non-synchronous process of shared intentionality. A central argument is that both infants and social relationships develop through transformative “moments of meeting,” which are intersubjective moments occurring between social partners, that can lead to re-organization of both the relationship itself, as well as the individuals’ implicit procedural knowledge about ways of being with others (Stern et al., 1998). A related theme focuses on the role of emotion, arguing that they contribute to mutual engagement with other emotional beings, due to both evoking and provoking further emotions in others (Reddy, 2019). Moments of mutual engagement, sometimes referred to as “encounters with other minds,” are seen as transformational. They allow us to be “seen,” or “known” by others, and therefore allow us to develop into complete persons (Reddy, 2019).

Within these frameworks, there are many research examples that highlight how essential psychological connectedness is for individual development. We summarize two that have been particularly well established and influential. The first example is the institutionalized children in Romania in the 1980’s (Nelson et al., 2007), many of whom died under conditions of adequate physical care due to lacking psychological care. This horrific example demonstrated to the world the unconditional human need for psychological closeness and provoked widespread research efforts to extend our knowledge about the implicit processes underlying this life supporting nearness. The second example is the Still Face paradigm (Weinberg and Tronick, 1996), which is an experimental setup where the infant and caretaker first interact in an everyday playful way and then the caretaker suddenly freezes and does not interact at all. This procedure typically affects the infant (and viewers of the experiment) immediately with almost unbearable distress. The Still Face paradigm reliably demonstrates the extreme aversiveness that accompanies social disconnection. It has also been widely used to study the high-precision temporal micro-adjustments that are present in normal interaction, with results showing that both infants and caretakers mutually contribute to regulating the interaction (De Jaegher et al., 2010).

Based on findings from the Still Face paradigm, Tronick and Beeghly (2011) formulated the Mutual Regulation Model (MRM) for understanding both individual and relational development. The notion of Social Breathing is closely connected to this research, representing the process of mutual regulation occurring in social interaction and suggesting the individual capacities that are necessary for it. The MRM connects meaning making to the idea of a “*core biopsychosocial state of consciousness*,” which represents infants’ non-verbal “meanings” (e.g., their affect, visual representations, etc.) about themselves in relation to the world, which shapes their ongoing engagement with the world. It is this state of consciousness that is co-created together with others through mutual engagement and becomes the basis for shared experiences and meanings (Damasio, 1999; Gottlieb and Halpern, 2002). The MRM takes a dynamic systems perspective and argues that this process involves dynamic self-organization, leading to new systemic properties, such as individual language development for infants, which in turn shapes new meanings as the child becomes capable of linguistic representations (Tronick and Beeghly, 2011). This developmental process has been described by using the rain-metaphor, where shared moment-to-moment interaction may be seen as raindrops that sculpt the landscape, or form the shared states of consciousness that are possible. The landscape will develop pathways, or chronic meanings, which constrains where the water or social interaction may flow in the future (Granic and Patterson, 2006).

The MRM extends the biopsychosocial view of an individual as a system to encompass the dyad with its own systemic properties. Specifically, the MRM suggests that two individuals form a dyadic system, in which they experience *dyadic states of consciousness* and co-regulate in ways that maintain homeostasis together (Tronick, 2007). This dyadic regulation will have two sides of scaffolding. On the one side, dyadic processes will shape the individual’s implicit understanding of *ways to be together*, meaning the actions, states and experiences that are allowed, or possible, within the relationship. On the other side, the process will continuously reshape and develop the dyad’s resources and abilities. The overall effect is that the individuals and the relationship continuously develop toward more complexity and deeper meaning making. In keeping with the MRM, Krueger (2013) argues that certain early experiences are jointly owned by the infant and caregiver and refers to this as the “joint ownership thesis.” Drawing upon both Merleau-Ponty’s phenomenological analysis, as well as studies of attention and mutual affect regulation, he argues that the phenomenological structure of some early infant–caregiver dyadic exchanges, such as the positive emotions that arise within these early exchanges, are jointly owned and cannot be allocated to one or the other individual separately (Krueger, 2013).

The MRM also takes a stance against the common use of simple synchrony (e.g., simultaneous actions or occurrences between social partners) as the focal measure of relational processes (Tronick and Beeghly, 2011). The authors of the MRM point out that empirical evidence does not support the idea that synchrony is central to individual and relational development, but rather presents a view of the co-regulative process as “messy,” an ongoing flow of missteps, matches, tries, retries and match-ups. Partners move continuously from matching states of shared intentionality to mismatched states and back, clearly showing the need to go beyond simple synchrony and “tidy” linear processes, at least if one wishes to understand the dynamics of relational systems, including processes such as cooperation and the development of group culture (Reddy and Uithol, 2016).

Going beyond dyads, every social context involves a culture of shared beliefs, norms and a meaning-system that is changing over time due to the members’ implicit sharing of mental content. Culture often refers to shared knowledge structures within large collectives of people, but culture can apply in small contexts such as a family or a group at the workplace (Wheelan, 2005). Tomasello (2014) puts culture in evolutionary terms and refers to “essential human pre-conditions.” His studies show that the ability to engage in shared intentionality is a pre-condition for collaboration, shared goals and creating a culture, and that it is this ability that allows humans to do all our obvious high-impact human behaviors (for good and bad). These unique abilities to act with shared intentionality and to understand the world as a plural subject are assumed to be a result of evolutionary demands for coordinated social behavior to survive as a group (Dunbar and Shultz, 2007; Tomasello et al., 2012; Tomasello, 2014).

The notion that an interwoven communicative process is involved in cultural development is also found in research on parent-child or therapist-client relational systems, and is often referred to as the capacity for dyadic consciousness (Tronick et al., 1998). Dyadic consciousness is what the persons in the dyad assume can be done within the relationship, e.g., what does any given behavior mean, which actions will lead to a disaster, and which expressions are accepted? A key feature is that the development of relational possibilities, or the dyadic culture, is shaped by an interwoven implicit process, requiring each individual to have the ability to engage in the multi-person system. Thus Social Breathing can be seen to be a fundamental process involved in the development of individuals, relationships and group culture.

## What Research on High Functioning Autism Can Teach Us About Social Breathing

Autism spectrum disorders (ASD) provide a natural context for understanding individual social abilities, and more generally, interpersonal processes and their neural underpinnings (Du Bois et al., 2014). From a relational perspective, the main disabilities for persons with high functioning autism (HFA) are understanding implicit material in interaction, co-constructing meaning, and developing the social skills that are taken for granted among neurotypical persons (Hobson et al., 2013; Di Cesare et al., 2017; Larkin et al., 2017). All three of these are related to our notion of Social Breathing, suggesting that the difficulties associated with HFA may contribute to disrupted Social Breathing when individuals with HFA attempt to interact with others.

There is no consensus on a theory to explain the underlying mechanisms in HFA (Khalil et al., 2018). Theories have pointed out different key aspects, but social, motor and sensory processes seem to be interconnected to a higher degree than previously thought (Bolis et al., 2017). Two of the primary approaches focus, on the one hand, on *active engagement* (Zahavi, 2011; De Jaegher, 2013), versus a more *cognitive* Theory of Mind (ToM) on the other hand (Baron-Cohen, 1999). The former emphasizes the ability to be engaged in face-to-face social interaction and to perceptually access and understand the intentions and experiences of others in a relatively direct way (Gallagher, 2001; Krueger, 2019). This approach argues that inner states are not entirely opaque and that observed behavior, in combination with context, provides a low-level route for accessing others’ minds (Zahavi, 2011). In contrast, the ToM approach emphasizes mentalizing, where the focus is on the ability to abstractly acquire an understanding of the social world by purposefully creating cognitive representations of other’s inner states (Baron-Cohen, 1999; Goldman, 2012). A related, but not identical approach, emphasizes embodied simulation, whereby unconscious modeling of our own acting body enables us to implicitly understand the actions performed by others, and to directly decode their emotions and sensations by simulating what we would experience in their context (Gallese, 2005). Our concept of Social Breathing strongly aligns with the former engagement approach, leading us to argue that understanding and treating HFA would benefit from a focus on the neural bases of disrupted engagement (e.g., the ability to engage in Social Breathing), rather than to *cognitively understand* others, either through theoretical inference (ToM) or imaginative projection (simulation).

Another relevant finding is that people with HFA tend to be poor at intersubjective coordination, which is at the very core of social engagement and interaction with cognitive, motivational and affective aspects (Hobson et al., 2013). The authors of the Interactive Brain Hypothesis (Di Paolo and De Jaegher, 2012) point to this evidence and suggest that it is not deficiencies in cognition or motivation that lead to problems in sharing implicit material, but rather that the problems in sharing implicit material lead to deficient cognitive and emotional development. In other words, problems with engaging in Social Breathing appear to be central to cognitive and motivational deficiencies in HFA, rather than the other way around.

There is also evidence that persons diagnosed with HFA suffer from motor deficits that undermine their ability for social interaction (Wimpory et al., 2002). For example, research suggests persons with HFA have a reduction of Purkinje cells, which explains their difficulties coordinating fine motor movements. In turn, this motor deficit sabotages preverbal social coordination and the use of proto-conversations, which are highly time-sensitive (Wimpory et al., 2002). One study using conversation analysis revealed that lower levels of social engagement in people with HFA was not correlated with general language ability, but rather with the ability to maintain the flow of moment-to-moment interpersonal engagement (Larkin et al., 2017). Similarly, other work has shown that persons with HFA have difficulty in adopting the bodily psychological stance of other persons, i.e., being moved affectively by the other (Hobson and Hobson, 2007) and that during a social interactive task, a higher level of HFA traits predicted less ability to modulate joint action. During a non-social task, however, HFA traits did not predict differences in movement coordination, thus pointing to the inherently social nature of the deficits (Curioni et al., 2017).

The findings above suggest that there are individual neurological differences in the ability to take part in implicit relational processes, but there is no consensus about how to study these processes, or what it means with respect to relational development. Arguably, relational understanding is not mainly about general language ability or intelligence, but instead is connected to the ability to engage in a mutual process of sharing meaning, or in other words, an ability for taking part in Social Breathing. Any disability in this domain is in turn connected to problems in the development of social competence, which may be described as “ways of behaving,” i.e., “how do you say hello to someone you haven’t met before, but is the friend of your friend?” or “how do you end a meeting without saying that the meeting is ending?” Neurotypical persons take such skills for granted, or at least learn without deliberate practice. Thus we suggest that focusing research on the aspects of HFA that contribute to disrupted Social Breathing could be a way forward in developing both reliable early assessment and valid feedback training programs, while at the same time advancing our knowledge of the mechanisms of social engagement.

## What Research on Psychotherapy and Medical Empathy Can Teach Us About Social Breathing

Empirical research and theoretical developments in psychotherapy generally focus on predictors of positive therapeutic outcomes for the patient. But research in this domain also provides a window for understanding the dynamics of human cooperation. Helpfully, compared to many other relational areas, the goals and structures of psychotherapy are well defined and there is a large amount of research. In addition, compared to other relational ventures, there is a strong focus on evidence-based practice. Still, drawing conclusions is scientifically difficult due to the large number of uncontrollable confounding factors. For example, psychotherapy often occurs 1 h a week, while at the same time a continuous stream of outside influences affects the patient’s symptom development. What is known is that psychotherapy makes a difference for people, but there is less consensus about the mechanisms of the change-process.

What seems clear is that there are some common factors that produce positive change across different forms of psychotherapy (Lambert and Barley, 2001) and the most influential is the quality of relationship between patient and therapist (Lambert and Barley, 2001; Horvath et al., 2011; Laska et al., 2014; Norcross and Lambert, 2014). But why might this be the case? Naturally, therapy takes place in a relationship between a therapist and a client, and an effective working relationship is necessary in a fundamental sense. But in what way is the quality of the relationship an important part of psychotherapy? A number of different approaches have been taken to address this question. One is to use self-report questionnaires based on concepts such as Working Alliance (Bordin, 1979), where patients and psychotherapists provide self-reports about the relationship, including the goal of therapy, tasks (e.g., how will we do this therapy? who does what?) and the patient’s and therapist’s emotional bond. Although this approach provides correlational evidence connecting relationship quality to therapeutic outcomes, it does not speak to mechanism and hence has limited utility for improving therapy or training therapists.

In addition to self-reports, methods from relational psychophysiology have been used to investigate the therapeutic relationship, often with a focus on physiological synchrony (Marci et al., 2007; Ramseyer and Tschacher, 2014; Karvonen et al., 2016; Tschacher and Meier, 2019). Unfortunately, this work lacks overarching theory, and its findings have been characterized as fragmented (Palumbo et al., 2017). One attempt to create a theoretical frame is the In-Sync model (Koole and Tschacher, 2016), which starts from the assumption that the alliance between patient and therapist emerges from the coupling of neural activity. Put in simple terms, the more tightly coupled, the better the alliance. The logic behind this is that low level perceptual-motor coordination between therapist and client supports a successful working alliance at the level of shared tasks and affect, including affective co-regulation, which in turn promotes patients’ adaptive emotion regulation and ultimately growth and healing. This model considers synchrony between therapist and patient (e.g., partners moving in a synchronized way over time) at three levels differing in both cognitive complexity (perceptual-motor, complex cognition, emotional regulation) and in temporality. The first temporal scale is the *phasic* time-scale from less than a second to about 10 s, with automatic associations and simple forms of cognitive inferences taking place. The second level is called the *tonic* time-scale, from 10 s up to an hour, with more complex social cognition and reasoning emerging. The third level is the chronic time-scale, from several weeks to years, during which higher-order abilities develop, such as effective emotion regulation.

Although the In-Sync model is a substantial step forward in thinking about the therapeutic alliance, we argue that its focus on synchrony is a limitation and that to understand relational systems we will need to consider more complex patterns of interpersonal coordination (Butler, 2011; Reed et al., 2015; Reddy and Uithol, 2016; Mu et al., 2018). For example, the different levels of the system (e.g., perceptual-motor, cognitive, affective) may coordinate between partners at different time lags, or at different frequencies of oscillation, or with different degrees of in-phase versus anti-phase patterning. As another example, in the affective domain it is relatively rare for two people to be in the same emotional state. More often, partners experience complementary states, such when one person gets angry and the other responds with fear or submission (Zahavi, 2011). Similarly, infants are able to respond appropriately in a coordinated way to parental behaviors that the infant is not yet capable of performing themselves (Reddy and Uithol, 2016) and a similar situation may arise for therapists and clients. In summary, the overall dynamics of the therapist-client system are likely quite complex, involving both shared, similar trajectories for some components, but complex and contrasting behaviors in other domains (Hari, 2017). We thus argue that extending the In-Sync model to address this complex temporal patterning may be a useful way to advance our understanding of the interpersonal coupling involved in therapeutic processes.

Another theoretical approach to understanding the therapist-client alliance is the model of Real relationships (Gelso, 2009). This model does not contradict the In-Sync model, but rather adds to it by stressing the automatic, implicit relational system in a way that directly corresponds to our notion of Social Breathing. The model of Real relationships suggests that there is a difference between the formal/technical elements of the therapeutic relationship and the more personal encounter (e.g., the Real relationship between patient and therapist) that in itself is argued to be curative, but difficult to distinguish from the professional one (Jones, 1968; Gelso, 2009; Kivlighan et al., 2016, 2017). Pre-requisites for a Real (personal) relationship are described as realism and genuineness. Realism concerns the ability to perceive the other with minimal interference from things such as projections, fears or wishes. Genuineness is in contrast to being phony, e.g., being authentic and true to oneself. A related set of ideas has been proposed by De Jaegher (2019), who discusses human knowing as a fine balance between the knower and the known, as they meet in the process of knowing-and-being-known. Knowing and loving are suggested to both be ways in which concrete and particular beings engage with each other, whereby the people involved need to deal with being-determined (by the other, by the relationship, and by themselves) and with determining (the other, the relationship, and themselves) (De Jaegher, 2019).

The Real relationships perspective highlights both the importance and the difficulty of willingly shaping and constructing functional and healthy relationships, both within a professional framework (psychotherapy, leadership) and in the private sphere (parenting, romantic partnerships). For example, a common situation that emphasizes the spontaneous and automatic nature of social interaction occurs when therapists find themselves unable to genuinely engage with a patient in a personal encounter, despite formal training and experience. We see this as a break-down in the therapist’s ability to engage in Social Breathing, suggesting that research in this domain could contribute to a better understanding of factors that inhibit spontaneous implicit social engagement.

Medical empathy is another domain involving research about change-processes, cooperation and health. Even though the amount of research in this field is not as vast or structured as in psychotherapy, recent conceptual discussions correspond quite closely to our construct of Social Breathing. In particular, there has been a recent critique of the un-humanistic and instrumental view of empathy within doctors training (Pedersen, 2010), where empathy is relegated to emotional contagion or cognitive simulation (Slaby, 2014). These views of empathy are argued to miss the point of “feeling with another” and getting access to another’s experiences (Slaby, 2014; Hooker, 2015). Critiques of this view argue for a more embodied approach to human knowing, a phenomenological account of empathy as social, interpretive and experiential, very closely connected to notions of intersubjectivity (Zahavi, 2015) and joint participation (Makkreel, 1996). This alternate model of medical practice involves a more experiential mode of understanding, where the doctor is moved by the patient’s experiences, rather than seeing it as a cognitive process (Halpern, 2003). Relatively extensive work within both medicine and philosophy argues for the need to take both the body and intersubjectivity seriously in medical practice, with the aim of getting both doctors and patients to understand illness in a broader sense than is usual in biomedicine (Hooker, 2015). This discussion is in complete accord with our model of Social Breathing, emphasizing that human social understanding is a shared, implicit, automatic and temporal venture. Further, by extension, Social Breathing requires individual abilities for engaging in relational systems, abilities that should be a prerequisite for a person’s empathic capacity, including in medical practice.

In summary, we argue that psychotherapy and other curative relationships such as doctor-patient relationships have their curative effects partly due to a multi-person process where the therapist/doctor is engaged in dyadic sharing of intentionality with the client. Thus successful healing requires both the healer and the client to have the individual abilities necessary for engaging in Social Breathing. Further, research within the frameworks of psychotherapy and medical empathy could be important for understanding relational processes in general, as well as for promoting better psychotherapy and medical practice.

## Studying Multi-Person Whole-Brain-Body Networks to Understand Relational Systems

Given the centrality of automatic, implicit, dynamic mutual sharing of social content for human functioning, it must have an evolved neurological basis. Research in this domain has seen a recent shift from a single-brain to a multi-brain frame of reference (Hasson et al., 2012; Schilbach et al., 2013), a shift that has been referred to as “second person neuroscience” (Caruana et al., 2017). At the heart of the shift is growing evidence that there is a fundamental difference in brain activity when people perceive themselves as being engaged in interaction (second person) compared to observing interaction (third person) (Redcay and Schilbach, 2019). This is also discussed as the dual nature of social cognition, with observing and participating as different mechanisms (Konvalinka and Roepstorff, 2012). Another related theoretical framework is the “interactive brain hypothesis” (IBH) (Di Paolo and De Jaegher, 2012). The IBH argues strongly against the idea that mindreading is the crucial part of social cognition and instead emphasizes a more enactive approach. The idea is that interpersonal coordination dynamics are the basis of social understanding, not the other way around. The IBH suggests that the evolutionary drive for minimizing cognitive load has selected for the more automatic process of understanding others through active participation in multi-person dynamical systems, as opposed to the more costly computing of the “mental state” of others (De Jaegher et al., 2010; Redcay and Schilbach, 2019).

Although second-person neuroscience is a notable step forward in understanding relational systems, there are a number of remaining limitations. One is that the second person perspective typically stops at the dyad and ignores triads or larger social groups, yet the shared “we” that can emerge has been argued to be crucial for explaining intentionality in these larger groups as well (Moore and Paulus, 2013). A second critique, more specifically aimed at social neuroscience, is that much of the research suffers from a reductionist approach and naïve scientism that risks missing the target due to disassembling social coordination into its smallest parts (Cummins, 2013). This critique becomes salient as the field moves toward a more active-engagement perspective, which highlights the need for dynamical modeling and systemic perspectives that view the dyad or larger social group as a single system (Di Paolo and De Jaegher, 2012; Cummins, 2013).

Perhaps the most important critique of social neuroscience is that the rapidly growing body of empirical findings has not led to a corresponding advance in our understanding of the social brain, mostly due to the lack of an adequate theoretical framework (Hari et al., 2015). One example of this problem is the emphasis on mirroring and synchrony, which are likely overly constrained for understanding complex interaction dynamics (Reddy and Uithol, 2016; Redcay and Schilbach, 2019). A related critique is that we may need to re-evaluate the Mirror Neuron system, since based on the logic of the Interactive Brain Hypothesis it may be epiphenomenal. In other words, the mirror neuron system may be in play during social interaction, but it may not be at the core of the relational process (De Jaegher et al., 2010). Indeed, essentially all the evidence regarding mirror neurons comes from single person experimental designs that cannot speak to actual social interaction (Mu et al., 2018). Further, if we accept the argument that the core of relational processes involves dynamic interpersonal coordination (Froese and Gallagher, 2012), then the idea of mirroring is simply misleading, since what we are trying to understand is a complex process requiring at least two distinct interaction partners, each playing their own unique role in the evolving relational system. Mirroring and synchrony miss this distinctness by over-emphasizing similarity between social partners (Reddy and Uithol, 2016).

Methodologically, research on interwoven social interaction will require a focus on naturalistic social interactions that are realistically complex, with open-ended tasks and simultaneous multimodal recordings (e.g., assessing brains, biology, and behavior) of engaged participants (Hari et al., 2015; Caruana et al., 2017; Redcay and Schilbach, 2019). Further, studies should be designed around falsifiable hypotheses about how multi-person systems work (Hari et al., 2015). The study of Social Breathing should also focus on the interwovenness of social partners, rather than considering only push-pull or leader-follower patterns. In other words, we need to think beyond mirroring and synchrony (Hasson and Frith, 2016) and take the shared process into account as *something else* than the tennis match metaphor of sending and receiving (Hari, 2017).

Another issue is that at the neural level we will need to take a whole-brain approach. A glance at the literature on social neuroscience shows that most studies set out to find a specific brain area (or perhaps a few areas) associated with some social process of interest. The list of areas generated by these studies ends up covering the whole brain, a result that is in line with the work on the “social brain atlas,” that concludes that there is no region, or even network, that is devoted only to social processes (Alcala-Lopez et al., 2018; Redcay and Schilbach, 2019). Just to show a few examples, there is work associating different brain areas with decoding of language (Lescroart and Gallant, 2019), eye-to eye interaction (Hirsch et al., 2017), communication by drumming (Rojiani et al., 2018), joint action coding (Ferrari-Toniolo et al., 2019), intersubjectivity (Vogeley, 2017), emotions promoting brain synchronization (Nummenmaa et al., 2012), face-to-face communication (Yun, 2013), ToM (Gallagher and Frith, 2003), inter-brain synchronization (Dumas et al., 2010), social perception (Deen et al., 2015), cross-brain mechanisms for verbal communication (Hirsch et al., 2018), and anthropomorphic bias (Chaminade et al., 2007, 2015). Clearly, future research will need to emphasize functional coupling across the entire brain, rather than trying to dissect social processes into isolated brain areas.

In addition, we will need to go beyond the whole-brain of the individual to encompass the whole-person (including brains) of multiple interacting social partners. In other words, we will need to take a multi-person whole-brain-body approach. Research using multi-brain hyperscanning has been increasing in recent years and has found stronger associations between coupling patterns across brains and relational processes than have been found with individual intra-brain markers (Mu et al., 2018). Hyperscanning work has not typically included other measures of the people involved, however, which might be important for understanding how multiple people become one coordinated system. For example, measures of biological and behavioral coordination may help to understand the complex dynamics underlying interwoven social engagement. In line with this, we propose that research should focus on the fact that the innate ability to engage in relational systems is primarily serving the goal of enabling the dyad or group system to continuously maintain the shared intentionality necessary for complex cooperation, rather than serving some individual-level function (Tomasello et al., 2012).

Finally, the concept of “Vitality Forms” (VFs) may prove useful in studying Social Breathing. VFs are a concept that highlights the motor-dependent, dynamic and temporal aspects of our ability for engaging in social systems, and thus descriptions of VFs suggest key individual capacities necessary for Social Breathing to occur. The term was coined by Stern (1990, 2010) in an effort to conceptualize the process and biological underpinnings behind shared subjectivity and shared intentions, i.e., intersubjectivity. VFs concerns the dynamics of movement, its pattern in force, directionality, time and space. When putting down a cup of coffee after a sip, is it in a sloppy and nonchalant way, a slow and precise way, or a hasty and aggressive way with a slamming sound? VFs are related to the idea of Affective Atmospheres, which can be thought of as a spatially diffuse version of VF’s (Anderson, 2009). VFs appear in writings on neuropsychology (Di Cesare et al., 2014, 2016), psychotherapy (Stern, 2010) and autism (Rochat et al., 2013; Gallese and Rochat, 2018; Krueger, 2019). Studies of VFs have shown differences in the neurological underpinnings of understanding “what is being done” from “how something is being done” (Di Cesare et al., 2014), and it is argued that VFs are related to a specific neural system that handles much of the implicit and affective information we pick up about the inner states and dispositions of other people (Ammaniti and Ferrari, 2013; Gallese and Rochat, 2018; Marraffa and Meini, 2019). Thus the ability to recognize and understand VFs should be closely related to the ability to engage in Social Breathing.

One way of getting a clearer view of VFs and their relation to shared intentionality is to consider the performing arts, e.g., dance, music, theater, and movies. These areas exemplify the deliberate and precise use of otherwise automatic processes to impact the experience of an observer (Stern, 2010) and to create Affective Atmospheres that permeate the collective situation, yet can be felt as intensely personal (Anderson, 2009). VFs are carefully modeled by the choreographer who instructs the dancers’ subtle movements in space and time, by the composer when coordinating melancholic melodies, and by the director who modulates tension between one scene and another. Movements that are modulated to communicate the intent of the actor allow the public to directly experience implicit social and emotional content. For example, when an actor conveys to the audience that the impersonated character wants to be appreciated, VFs are a key part of this expression of intent. Thus VFs may provide an objectively measurable and modifiable indicator of the implicit content being exchanged during Social Breathing, which would make them a useful target for research attempting to unpack both the interwoven exchange of Social Breathing itself, as well as the individual capacities necessary to engage in and sustain it.

## Measuring Social Breathing

**FIGURE 2:**
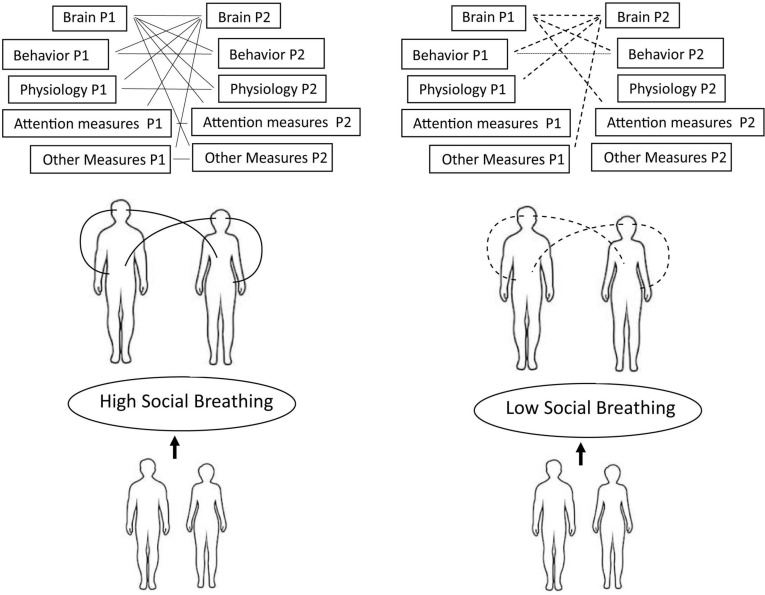
A proposal for measuring the latent construct of Social Breathing. We suggest that Social Breathing can be measured during temporally contingent social interactions using repeated measures of the multi-person whole-brain-body system. Higher Social Breathing is indicated by a denser network of connections among the observed measures, which would support the emergence of more complex forms of interpersonal coordination. In contrast, lower Social Breathing would be indicated by fewer or weaker connections among the measured nodes in the network, as indicated by the reduced number of connections and the dotted rather than solid lines in the figure.

Figure 2 shows an overview of our proposal for how to measure Social Breathing, which we consider to be a latent construct (e.g., a theoretical entity that cannot be measured directly). To begin, we suggest that it can only be measured during temporally contingent social interaction, such as face-to-face conversations or electronically mediated interactions that allow real-time coordination between partners. In Figure 2 we show a two-person version, but the model can expand to include any number of people in contingent interaction. Although such temporal contingency is necessary, it is not sufficient. Social Breathing may, or may not, emerge in such interactions. For example, one person may not have the neurotypical skills or the motivation to engage in Social Breathing, in which case it would not emerge.

At an intermediate level of abstraction, we propose that when Social Breathing emerges it can be observed as co-ordination between the brains and bodies of a multi-person system. Ultimately the brains are driving the process, but people can only become interwoven with each other via the rest of their body, since it primarily via the observables of the body that we can “touch” each other. Thus we need to take both brains (the control centers) and the rest of the bodies (the physical interface between partners) into account. At the level of more specific measures, there are many possible indicators of a multi-person brain-body system, so long as they are measured with fine temporal resolution from all the people involved. We have emphasized: (1) multi-site EEG and fNIRS as indicators of whole-brain activity, since Social Breathing involves coordination across many neural sub-systems, (2) measures of the peripheral nervous system, such as heart rate and skin conductance, since Social Breathing involves coordination of arousal, (3) eye-tracking as an indicator of attentional focus, since Social Breathing involves coordination of joint attention, (4) VFs, assessed from movement, speech characteristics (e.g., pitch) and facial expression, since VAFs convey much of the implicit and affective information that is automatically exchanged during Social Breathing, and (5) explicit communicative acts assessed from language, gestures and facial expression, since when available, Social Breathing can be facilitated by these more conscious channels.

You are not done measuring Social Breathing once you have data, however. It also requires a mathematical model of complex multimodal, hierarchical coordination across all the measures. In other words, Social Breathing is not indicated by the measures themselves, but by the temporal coordination among them. As shown in Figure 2, we propose that higher Social Breathing is indicated by a denser network of connections among the observed measures, which would support the emergence of more complex forms of interpersonal coordination. In contrast, lower Social Breathing would be indicated by fewer or weaker connections among the measured nodes in the network. It is also important to note that “higher” Social Breathing does not imply a better quality relationship. We can become deeply interwoven with others during arguments (e.g., Social Breathing is at a high level), even though we might consider the relationship to be a destructive one. A central challenge moving forward, therefore, will be to develop appropriate dynamic models that can represent the complexity of the interwoven multi-person whole-brain-body interaction that we refer to as Social Breathing.

Critically, in reviewing the literature we found relatively few empirical examples of applying dynamic mathematical models to data for looking at interwovenness of the *dyad or social group as a whole*. Most of the examples we did find were from studies of family relationships (Hollenstein and Lewis, 2006; Butner et al., 2007; Butler, 2011; Helm et al., 2012; Ferrer and Helm, 2013; Reed et al., 2015) or psychotherapy within the framework of the *Real relationship* model (Peluso et al., 2012). So far, relevant research using brain hyperscanning methods has relied on the simplest measures possible, such as synchronicity or single-channel coupling (Liu et al., 2016; Mu et al., 2018). In addition, coupling or Granger-Causality (Schippers et al., 2010) are typically only applied to small pre-selected sets of neural indicators (e.g., one or two EEG frequencies, or one or two fNIRS locations) and have not been extended to consider multi-person whole-brain-body networks (e.g., combining multimodal measures of behavior, physiology and neural functioning from multiple interacting people).

Importantly, however, a number of research groups are taking a dynamic systems perspective and are actively developing models appropriate for assessing emergent system behaviors such as Social Breathing. For example, one suggestion is to make use of the Probabilistic Graphical Models (PGM) framework (Koller and Friedman, 2009), where one can explicitly model both dyadic (or larger social group) multivariate dynamic processes, such as Social Breathing, and individual processes contributing to that emergent group process. In one example from our own work we used a Generative Bayesian Coupled Linear Oscillator model to investigate emotional dynamics in romantic couples and showed that individual health indicators predicted different couple-level emotion dynamics during face-to-face interaction (Guan et al., 2015). Another example is the work of Kelso and colleagues who have developed an extended version of the Haken-Kelso-Bunz (HKB) model representing both the intrinsic behavior of a system (e.g., how it would behave if not influenced by outside forces) and constraints on system behavior arising due to interacting with another unit (Tognoli et al., 2020).

A number of other models have been developed in the context of studying speech and conversation. For example, Abney et al. (2014) have applied Power Law models to investigate language during social interactions. A power law function expresses one variable as a non-linear function of another variable raised to a power, which implies that variability occurs across different scales of measurement. Power law functions are known to apply to complex systems with hierarchically nested structures and processes. Abney et al. (2014) showed that social partner’s power law functions became more similar during affiliative social interactions, a process they call complexity matching. Another approach has focused on “synergies,” which refers to coordination in a system arising through a reduction in the degrees of freedom as social partners adapt and attune to each other (Dale et al., 2013; Fusaroli et al., 2014). Temporal network models have been suggested for studying synergies, where increased coordination would be represented by the connections between nodes becoming less random, stronger and more stable (Dale et al., 2013). In summary, a number of promising models are being developed, but so far they have not been applied to multivariate data from interacting social partners, which represent a critical next step in the study of Social Breathing.

## Future Research Directions Based on the Social Breathing Model

Advancing our understanding of Social Breathing could have impact across a number of domains. For example, within autism research, a more integrated theory and measures may spark the development of knowledge necessary for assessment and feedback, with consequences for early detection of HFA, as well as evaluation of training programs. The situation is similar for psychotherapy and other psychological treatments. Given that the quality of the therapist/client relationship is one of the most important factors for successful psychotherapy, better objective data relevant to relational processes may prove fruitful for providing higher quality feedback during training. This is especially important since this lack of feedback is one reason that therapy sometimes does not work and that therapists do not get better over time (Macdonald and Mellor-Clark, 2015). Studies of Social Breathing could also lead to developments in human-computer interaction. There are numerous interactive interfaces, including systems based on knowledge about emotions, motivation, reinforcement, perception, cognition and health. But despite the large amount of research on AI, there is to our knowledge still no functioning social AI. Our research team is currently investigating whether knowledge about implicit interwoven interaction, gained from hyperscanning work with human team members, could help us develop an artificial agent that is better able to learn and communicate about humans’ shared assumptions and intentions, as well as predict both individual and group behavior. One application of socially intelligent software would be enhancing web-based therapy for a variety of mental health problems, a field that is growing in quantity and quality. Other obvious applications are the AI-assistants, such as Siri and Alexa, who are becoming more important for a variety of groups in education, in business and in everyday life both for healthy persons and for persons with mental and physical disabilities.

In summary, we propose that Social Breathing is a mutual, automatic, implicit and temporal multi-person whole-brain-body process that could be studied by focusing on coordination across multiple measures of partners engaged in real-time interaction. Specifically, higher levels of Social Breathing would be indicated by more complex, dense and wide-spread connectivity between the brains, bodies and behavior of social partners. These real-time processes could then be related to longitudinal investigations of the role of Social Breathing in the development of both individuals and relationships, as well as intervention studies targeting improved Social Breathing either in professionals (e.g., therapists in training) or those with deficits (e.g., individuals with HFA). Such methods would allow for testing falsifiable hypotheses across a number of research domains, including clinical psychology, psychotherapy, AI and group-theory, to name a few.

We conclude by suggesting a small set of such hypotheses:

(1) The construct of Social Breathing will have greater explanatory power for predicting relational outcomes than multi-person synchrony and other linear models of social interaction.
(2) Social Breathing will be associated with the subjective experience of the interaction, with higher levels of Social Breathing associated with feelings of connection, engagement and being understood in the context of affiliative relationships, but feelings of intensity and being unable to disengage in conflicted relationships. In contrast, low levels of Social Breathing will occur when an interaction partner is psychologically occupied elsewhere, or when there is a time-lag in audio/video-facilitated interaction, and will be associated with feelings of loneliness or disconnection.
(3) The ability to engage in Social Breathing will distinguish neurotypical persons from persons diagnosed within the Autism spectra.
(4) Social Breathing in therapeutic relationships will mediate the connection between relational quality and therapeutic outcome.

## Concluding Remarks

The basic and specific human ability for developing group culture probably precedes the development of abstract cognitive processes and perhaps even language (Tomasello, 2014). Sociality is thus a central requirement for human brain function (Di Paolo and De Jaegher, 2012; Hari et al., 2015). Given that group culture and collaboration depend on the individuals’ abilities to collectively develop shared meanings, we argue that it requires the ability to engage in multi-person whole-brain-body networks, a process we call Social Breathing. It is a process that is mutual (e.g., only happens in interaction with other persons) temporally sensitive, mainly implicit and, similar to physical breathing, it is automatically active without conscious planning. We argue that systematic research on Social Breathing could provide a comprehensive picture of relational dynamics, in contrast to the present situation dominated by a plethora of detail-focused and fragmented social neuroscience results. Ultimately, this should lead to reliable and valid measures that correspond to both relational outcomes and individual experiences in relationships, allowing for a full-blown intervention science of relationships.

## Author Contributions

Both authors have been involved in initiating the original idea for the manuscript and took full responsibility for the text. NK has been mainly responsible for initial drafts, but both authors have been continuously working with analysis development and revising the manuscript.

## Conflict of Interest

The authors declare that the research was conducted in the absence of any commercial or financial relationships that could be construed as a potential conflict of interest.
